# The effect of zingiber officinale on prooxidant-antioxidant balance and glycemic control in diabetic patients with ESRD undergoing hemodialysis: a double-blind randomized control trial

**DOI:** 10.1186/s12906-023-03874-4

**Published:** 2023-02-17

**Authors:** Helya Rostamkhani, Parisa Veisi, Bahram Niknafs, Mohammad Asghari Jafarabadi, Zohreh Ghoreishi

**Affiliations:** 1 Student Research Committee, Tabriz University of Medical Sciences, Tabriz, Iran; 2 Department of Clinical Nutrition, Faculty of Nutrition and Food Sciences, Tabriz University of Medical Sciences, Tabriz, Iran; 3 Kidney Research Center, Tabriz University of Medical Sciences, Tabriz, Iran; 4 Cabrini Research, Cabrini Health, 154 Wattletree Rd, Melbourne, VIC 3144 Australia; 5 School of Public Health and Preventative Medicine, Faculty of Medicine, Nursing and HealthSciences, Monash University, VIC, 3800 Australia; 6 Road Traffic Injury Research Center, Tabriz University of Medical Sciences, Tabriz, Iran; 7 Nutrition Research Center, Department of Clinical Nutrition, Faculty of Nutrition and Food Sciences, Tabriz University of Medical Sciences, Tabriz, 5166614711 Iran

**Keywords:** Ginger, End-stage Renal Disease, Prooxidant-Antioxidant Balance, Insulin resistance, Diabetes, Blood glucose

## Abstract

**Background:**

Diabetes management in hemodialysis patients with end-stage renal disease needs precision to avoid complications. The study aimed to investigate the effect of ginger supplementation on prooxidant-antioxidant balance, glycemic management, and renal function in diabetic hemodialysis patients.

**Trial design and methods:**

Forty-four patients were randomly allocated to either the ginger or the placebo group in this randomized, double blind, placebo-controlled study. Patients in the ginger group received 2000 mg/d ginger for eight weeks, whereas those in the placebo group received equivalent placebos. After a 12- to 14-h fast, serum levels of fasting blood glucose (FBG), insulin, urea, creatinine, and prooxidant-antioxidant balance (PAB) were measured at baseline and at the end of the study. The homeostatic model evaluation of insulin resistance was used to determine insulin resistance (HOMA-IR).

**Results:**

Serum levels of FBG (*p* = *0.001*), HOMA-IR (*p* = *0.001*), and urea (*p* = *0.017*) were considerably lower in the ginger group compared to baseline, and the difference was significant when compared to the placebo group (*p* < *0.05*). Moreover, ginger supplementation decreased serum levels of creatinine (*p* = *0.034*) and PAB (*p* = *0.013*) within the group, but the effect was insignificant between groups *(p* > *0.05)*. On the other hand, insulin levels did not vary significantly across and among the groups *(p* > *0.05).*

**Conclusion:**

In summary, this study indicated that in diabetic hemodialysis patients, ginger could result to lower blood glucose levels, enhanced insulin sensitivity, and lower serum urea levels. Further studies with a more extended intervention period and various doses and forms of ginger are needed.

**Trial registration:**

IRCT20191109045382N2. (06/07/2020), Retrospectively registered, https://www.irct.ir/trial/48467

## Introduction

End-Stage Renal Disease (ESRD) is a severe form of kidney failure which may require dialysis or a kidney transplant [1]. Various factors may exacerbate renal failure, but diabetes has the most substantial influence [2]. In Iran, the prevalence of ESRD is predicted to rise by around 12% every year, with 48.5 percent of these patients getting hemodialysis [3, 4]. Between 2005 and 2021, the prevalence of diabetes in Iran's population expanded from 7.7% to 14.3%, and 29.2% of the population had prediabetes [2, 3, 5, 6]. This trend implies that the rise in ESRD cases in Iran is linked to the growth of diabetes.

ESRD, without a doubt, necessitates a certain treatment approach, but diabetes is another restriction that requires special care [2, 6]. Poor glycemic management in diabetic ESRD patients was shown to raise renal pressure and inflammation, worsen clinical status and increase mortality risk [1, 2, 6].

Regarding the importance of glycemic control in the patients suffering from diabetes and ESRD undergoing hemodialysis, the constraints in terms of kidney dysfunction, limited medical treatment pathways, and finding an economically effective medical supplement, which could cover both pathways, seems necessary [2, 7–9]. Among all methods of designing an effective medical supplement, herbal medicine seems to be the most popular method [10]. The evidence shows that more than 400 herbal medications, especially spices, could be helpful in the glycemic control of diabetes patients [10, 11].

One of these plants, ginger (Zingiber officinale Rosco), is thought to help manage glucose levels considerably [10, 12]. Ginger has various health benefits in addition to glycemic management, and it is widely utilized in traditional Chinese, Ayurvedic, and Tibb Unani medicine [13–15].

The antioxidant, anti-tumor, and anti-inflammatory properties of ginger are thought to be responsible for its health benefits [13, 14]. Ginger was indicated in many clinical investigations to improve fasting blood glucose (FBG), serum insulin, and insulin resistance [12, 16], as well as lower urea and creatinine levels [17–19]. Other evidence revealed that ginger might effectively treat renal disorders [20]. All of them, however, are still under study and will need more thorough research to validate or refute the present notion [20].

This study aimed to assess the effect of ginger supplementation on glycemic control, renal function, and prooxidant-antioxidant balance in patients with diabetes and ESRD undergoing hemodialysis, taking into account all of the potential benefits of ginger in glycemic control and improving health outcomes [13, 14, 21, 22].

## Materials and methods

### Study design

The current research was a randomized, double-blind, controlled parallel-group study including individuals with diabetes and ESRD who were on hemodialysis. The research was authorized by Tabriz University of Medical Sciences (IR.TBZMED.REC.1398.1188) and was filed on the Iranian Clinical Trials Registry website (IRCT20191109045382N2). Prior to intervention, all participants provided informed permission in accordance with the Declaration of Helsinki.

### Participants

**Fig. 1:**
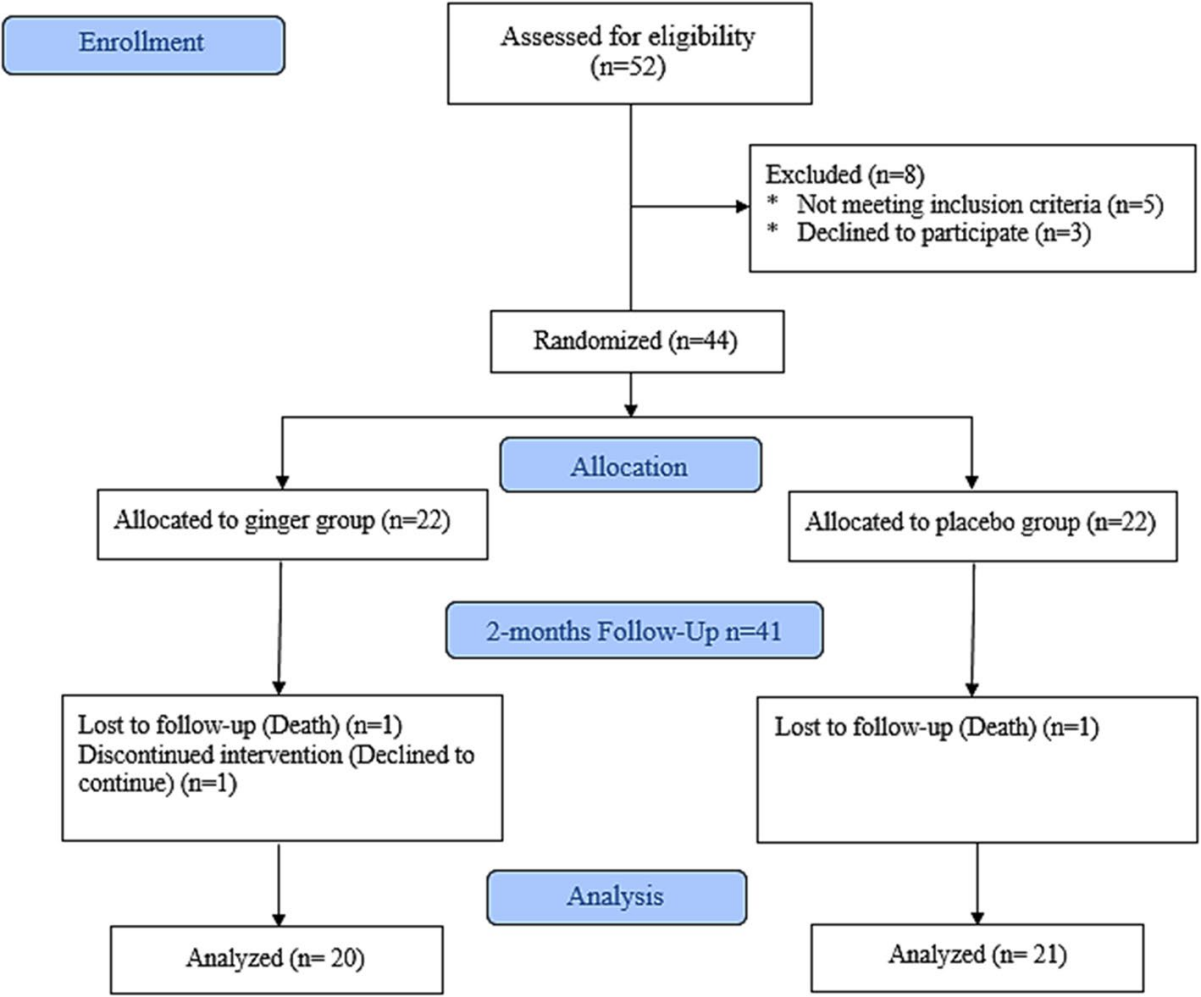
CONSORT Flow Diagram of the stud**y**

Between July 2020 and September 2020, Fifty-two (men and women) patients in the age range of years were recruited from the dialysis center of Imam Reza Hospital associated with Tabriz University of Medical Sciences in Iran. Patients who were 18 years old or older, diagnosed with T2DM, and received hemodialysis at least twice a week for the past three months were eligible for this study (each series 4 h). They also needed to be free of any acute gastrointestinal issues, thyroid abnormalities, gallstones, or a history of ginger sensitivity. After being accepted into the study, patients were ordered not to consume ginger for a month. They should not have taken fish oil supplements, steroidal and non-steroidal anti-inflammatory medicines, levothyroxine, warfarin, and antioxidant supplements. Exclusion criteria included altering kidney replacement treatment procedures (peritoneal dialysis or kidney transplantation) throughout the trial, inconsistent hemodialysis attendance, and refusal to continue supplementing. Throughout the research, a flow diagram based on the CONSORT declaration depicts the inclusion and removal of individuals (Fig. 1).

### Interventions

The participants were allocated into intervention and control groups via a randomized block procedure of size 2, stratified on age, gender, and FBS categories. The study statistician produced the random sequence with STATA 16 software. For eight weeks, patients in the ginger group took four capsules containing 2000 mg of ginger powder daily (Goldaroo, Co. Isfahan, Iran), whereas those in the placebo group had four placebo capsules containing starch. The ginger powder was weighed and packed into 500 mg capsules, whereas the placebo starch was packed into capsules with the same size, color, and odor as the ginger powder. The research executive and patients were unaware of the composition of the pills to maintain the study double-blind and assigning participants to interventions was done by someone other than the the research executive. All participants had laboratory blood tests at baseline and after the eight weeks following a 12- to 14-h fast. 7 mL of blood was taken and stored at room temperature (20–25C) for 20 min before hemodialysis. The samples were centrifuged at 2000 g for 10 min after clotting; serum samples were divided into tiny aliquots and stored at –70C until use.

### Measurements and outcomes

Physical activity was assessed using a modified short version of the International Physical Activity Questionnaire, whose validity and reliability had previously been published [23]. IPAQ was graded using established techniques [24], and the results were presented as a continuous measure in metabolic equivalent of task minutes each week [18]. At baseline and the end of week 8, the authors used a questionnaire to determine anthropometric indices such as weight, height, BMI, waist circumference, and hip circumference using standard techniques. Dietary intakes were measured using a 3-day dietary recall at the start and end of the experiment (2 days during the week and one day on the weekend). Nutritionist IV software (N-Squared Computing, San Bruno, CA, USA) was used to examine the patients' diets. Our primary outcomes were prooxidant-antioxidant balance (PAB), Fasting blood glucose (FBS), Serum levels of fasting insulin, and HOMA-IR. The serum levels of urea and creatinine were the secondary outcomes of the study. Insulin concentrations were assessed using enzyme-linked immunosorbent assay kit (Monobind Co. USA) [25], while FBG, urea, and creatinine concentrations were determined using commercially available enzyme kits (Pars Azmoon, Tehran, Iran). The homeostatic model evaluation of insulin resistance (HOMA–IR = [FBG (mg/dl) × fasting insulin (µu/ml)] **∕** 405) was used to determine insulin resistance. A prior approach established by Faraji-Rad et al. [26] was used to assess the PAB**.**

### Compliance

Each patient was given a certain number of capsules and told to return the unused capsules every week to ensure patient compliance. A somewhat varying number of capsules were returned at the end of the visits; nonetheless, compliance was satisfactory. The participants had more than 51 days of compliance during the intervention period, which means over 90% compliance in this study.

### Statistical analysis

The sample size was determined as 17 based on data values acquired from previous research with a confidence interval of 95 percent, power of 90 percent, and HOMA–IR as a key variable [12] using G*Power software v3.1.9.6. We scheduled 22 individuals in each group, assuming a 25% dropout rate.

Kolmogorov–Smirnov test was used to examine variable distributions, and according to the intention-to-treat principle, statistical analyses were done using SPSS version 21.0 (SPSS Inc., Chicago, IL, USA). Independent Samples t-test, Chi-square test, and Mann–Whitney U test were used to evaluate demographic and anthropometric data, physical activity levels, and dietary features of patients among research groups. To compare parameters among groups, paired samples t-tests or Wilcoxon signed ranks tests were used based on the normal data distribution. Endpoint analysis was performed on the intention-to-treat population using an analysis of covariance (ANCOVA), corrected for confounding factors, to see whether post-treatment outcomes in the intervention and control groups correlated with pre-test measurements. The R (4–2-2) programming language and its ggplot 2 package were used to create plots for the ANCOVA method. A *p*-value of less than 0.05 was deemed significant in all statistical analyses.

## Result

A total of 44 people aged between 31 and 77 years old were randomly assigned to either a ginger supplement (*n* = 22) or a placebo (*n* = 22) in a randomized placebo-controlled double-blind study. The intention-to-treat analysis included 41 of the 44 randomized patients. Table 1 shows the baseline characteristics of study participants; Obviously, there were no significant variations in age, gender, height, weight, BMI, dialysis frequency, or medicines (*p* > *0.05*).

**Table 1:** Baseline characteristics of study participants

Variables	Ginger group (*n* = 22)	Placebo group (*n* = 22)	*P-value*
Age (years)	60.05 ± 11.12	59.64 ± 10.69	0.902*
Sex			0.763†
Men (%)	11 (50%)	12 (54.5%)	
Women (%)	11 (50%)	10 (45.5%)	
Height (29)	161.46 ± 8.70	161.76 ± 9.62	0.916*
Weight (kg)	69.67 ± 10.76	74.55 ± 14.31	0.209*
BMI (kg.m^−2^)	26.48 ± 3.71	28.41 ± 4.35	0.123*
Dialysis Frequency			0.709†
Two sessions per week	4 (18.2%)	5 (22.7%)	
Three sessions per week	18 (81.8%)	17 (77.3%)	
Medication history			
Insulin therapy	5 (22.7%)	4 (18.2%)	0.709†

Values are reported as Mean ± SD or Median (IQR) for quantitative data, and frequency (percentage) for qualitative data

^*^Independent T Test, # Mann–Whitney U, † Chi-square test

### Energy and nutrient intakes, anthropometric parameters

At the start and the end of the trial, there were no significant variations in energy, macronutrient, and micronutrient consumption between the two groups and within group as well(*p* > *0.05*; Table 2; Table 3). Furthermore, there were no significant differences between the two groups in terms of weight, BMI, waist circumference, hip circumference, and physical activity levels at the baseline and after the intervention (*p* > *0.05*; Table 4).

**Table 2:** Comparison of mean energy intake and macronutrients in the study participants before and after intervention

Variables	Ginger group (*n* = 20)	Placebo group (*n* = 21)	Mean difference (CI 95%)	*P-value*
**Energy (Kcal/d)**				
Baseline	1628.95 ± 292.04	1646.77 ± 242.23	-17.82 (-181.23, 145.60)	0.827†
Endpoint	1650.55 ± 276.88	1644.02 ± 232.52	10.12 (-55.69, 75.94)	0.757††
Mean difference (CI 95%)	24.00 (-36.24, 84.24)	13.28 (-26.82, 53.38)		
P-value*	0.415	0.498		
**Carbohydrates (g/day)**				
Baseline	216.77 ± 41.20	219.30 ± 37.88	-2.52 (-26.60, 21.56)	0.834†
Endpoint	220.75 ± 39.04	219.92 ± 36.32	2.91 (-11.10, 16.92)	0.676††
Mean difference (CI 95%)	3.97 (-6.74, 14.69)	0.22 (-9.93, 10.36)		
P-value*	0.449	0.965		
**Carbohydrates (%Kcal)**				
Baseline	50.57 ± 2.54	50.53 ± 3.00	0.04 (-1.65, 1.73)	0.966†
Endpoint	50.35 ± 2.31	50.13 ± 2.44	0.22 (-1.30, 1.74)	0.772††
Mean difference (CI 95%)	-0.13 (-1.65, 1.40)	-0.35 (-2.38, 1.68)		
P-value*	0.863	0.722		
**Protein (g/day)**				
Baseline	52.42 ± 11.11	54.73 ± 9.11	-2.31 (-8.66, 4.04)	0.467†
Endpoint	52.78 ± 10.23	54.18 ± 7.65	-0.66 (-4.79, 3.47)	0.748††
Mean difference (CI 95%)	0.25 (-3.21, 3.71)	0.44 (-3.07, 3.95)		
P-value*	0.881	0.796		
**Protein (%Kcal)**				
Baseline	12.85 ± 1.71	13.38 ± 2.09	-0.53 (-1.69, 0.64)	0.367†
Endpoint	12.86 ± 1.73	13.27 ± 1.51	-0.24 (-1.12, 0.65)	0.592††
Mean difference (CI 95%)	-0.04 (-0.84, 0.76)	-0.02 (-0.84, 0.80)		
P-value*	0.925	0.957		
**Fat (g/day)**				
Baseline	63.17 ± 12.17	63.07 ± 12.26	0.09 (-6.78, 6.94)	0.979†
Endpoint	64.41 ± 12.13	63.94 ± 10.49	0.15 (-4.02, 4.33)	0.941††
Mean difference (CI 95%)	1.18 (-1.88, 4.24)	1.11 (-2.21, 4.43)		
P-value*	0.429	0.495		
**Fat (%Kcal)**				
Baseline	34.92 ± 2.71	34.46 ± 2.75	0.46 (-1.20, 2.13)	0.576†
Endpoint	35.19 ± 3.25	34.97 ± 2.80	0.10 (-1.76, 1.96)	0.911††
Mean difference (CI 95%)	0.19 (-1.28, 1.66)	0.34 (-1.36, 2.05)		
P-value*	0.792	0.678		

Values are reported as Mean ± SD. *Paired Samples T-Test, † Independent Samples T-Test, ††ANCOVA adjusted for baseline values

**Table 3:** Comparison of micronutrients intake in the study participants before and after intervention

Variables	Ginger group (*n* = 20)	Placebo group (*n* = 21)	Mean difference (CI 95%)	*P-value*
**SFA (g)**				
Baseline	15.72 ± 4.21	13.84 ± 3.94	1.87 (-0.61, 4.36)	0.135†
Endpoint	14.75 ± 3.14	13.15 ± 2.89	0.83 (-0.79, 2.45)	0.306††
Mean difference (CI 95%)	-0.34 (-1.56, 0.89)	-0.33 (-1.92, 1.27)		
P-value*	0.569	0.672		
**MUFA (g)**				
Baseline	15.86 ± 3.94	14.88 ± 2.65	0.99 (-1.06, 3.03)	0.336†
Endpoint	15.67 ± 3.27	15.26 ± 2.63	-0.31 (-1.41, 0.80)	0.578††
Mean difference (CI 95%)	-0.12 (-0.95, 0.70)	0.50 (-0.51, 1.50)		
P-value*	0.760	0.314		
**PUFA (g)**				
Baseline	25.95 ± 5.43	28.25 ± 6.70	-2.30 (-6.01, 1.41)	0.218†
Endpoint	27.70 ± 6.91	28.33 ± 6.78	0.98 (-2.69, 4.66)	0.592††
Mean difference (CI 95%)	1.78 (-0.77, 4.34)	-0.17 (-3.19, 2.85)		
P-value*	0.161	0.908		
**Cholesterol (g)**				
Baseline	201.51 ± 92.72	167.50 ± 65.32	34.01 (-14.79, 82.81)	0.167†
Endpoint	229.28 ± 86.84	170.22 ± 77.06	44.73 (-4.44, 93.89)	0.073††
Mean difference (CI 95%)	30.27 (-7.49, 68.04)	6.50 (-37.55, 50.55)		
P-value*	0.110	0.761		
**Fiber (g)**				
Baseline	10.90 ± 2.05	11.89 ± 2.18	-0.99 (-2.28, 0.29)	0.127†
Endpoint	11.37 ± 2.80	12.04 ± 2.34	-0.22 (-1.86, 1.41)	0.787††
Mean difference (CI 95%)	0.29 (-0.85, 1.43)	-0.90 (-2.82, 1.01)		
P-value*	0.599	0.338		
**Phosphorus (g)**				
Baseline	597.74 ± 215.21	552.68 ± 121.13	45.06 (-61.19, 151.32)	0.397†
Endpoint	543.51 ± 204.50	508.90 ± 109.00	1.98 (-72.23, 76.19)	0.957††
Mean difference (CI 95%)	-53.78 (-116.29, 8.74)	-38.65 (-95.90, 18.60)		
P-value*	0.088	0.174		
**Potassium (mg)**				
Baseline	1543.34 ± 390.00	1815.81 ± 503.23	-272.47 (-546.39, 1.46)	0.051†
Endpoint	1598.60 ± 400.03	1637.86 ± 347.30	87.91(-113.59, 289.40)	0.383††
Mean difference (CI 95%)	49.36 (-97.74, 196.46)	-178.01 (-380.62, 24.60)		
P-value*	0.491	0.082		
**Sodium (mg)**				
Baseline	1012.87 ± 728.88	1253.15 ± 669.49	-207.44 (-633.77, 218.90)	0.332†
Endpoint	1120.33 ± 720.76	1147.49 ± 530.65	18.08 (-367.66, 403.82)	0.925††
Mean difference (CI 95%)	89.16 (-293.59, 471.91)	-46.00 (-393.56, 301.56)		
P-value*	0.631	0.785		
**Vitamin A (µg)**				
Baseline	896.83 ± 544.52	955.40 ± 624.13	-58.56 (-414.94, 297.81)	0.742†
Endpoint	806.67 ± 398.69	860.14 ± 407.91	-50.31 (291.59, 190.96)	0.675††
Mean difference (CI 95%)	-107.01 (-395.35, 181.33)	-126.88 (-367.23, 113.47)		
P-value*	0.447	0.284		
**Vitamin C (mg)**				
Baseline	71.27 ± 21.31	99.32 ± 76.00	-28.04 (-62.01, 5.92)	0.103†
Endpoint	56.22 ± 28.18	79.95 ± 45.18	17.09 (-41.17, 6.98)	0.159††
Mean difference (CI 95%)	-12.26 (-27.36, 2.84)	-21.26 (-55.30, 12.78)		
P-value*	0.592	0.207		
**Vitamin E (mg)**				
Baseline	2.21 ± 0.87	2.16 ± 0.74	0.05 (-0.44, 0.54)	0.842†
Endpoint	2.30 ± 1.50	2.49 ± 1.33	-0.26 (-1.09, 0.58)	0.534††
Mean difference (CI 95%)	0.03 (-0.52, 0.58)	0.33 (-0.34, 0.99)		
P-value*	0.903	0.316		
**Zinc (mg)**				
Baseline	4.50 ± 1.14	5.25 ± 1.56	-0.76 (-1.59, 0.07)	0.073†
Endpoint	4.25 ± 1.24	4.79 ± 1.23	-0.22 (-0.93, 0.48)	0.521††
Mean difference (CI 95%)	-0.22 (-0.79, 0.35)	-0.37 (-0.99, 0.25)		
P-value*	0.425	0.229		
**Selenium (mg)**				
Baseline	65.68 ± 19.78	76.36 ± 21.72	-10.68 (-23.32, 1.96)	0.057†
Endpoint	63.50 ± 13.87	71.43 ± 15.58	-4.55 (-14.13, 5.03)	0.342††
Mean difference (CI 95%)	1.50 (-5.17, 8.17)	-4.76 (-16.15, 6.63)		
P-value*	0.643	0.394		
**Magnesium (mg)**				
Baseline	125.64 ± 26.17	141.77 ± 30.99	-16.13 (-33.58, 1.32)	0.069†
Endpoint	114.41 ± 33.75	137.46 ± 29.48	-15.90 (-36.25, 4.47)	0.122††
Mean difference (CI 95%)	-12.66 (-31.47, 6.16)	-12.63 (-27.91, 2.65)		
P-value*	0.175	0.100		

Values are reported as Mean ± SD. *Paired Samples T-Test, † Independent Samples T-Test, ††ANCOVA adjusted for baseline values

**Table 4:** Comparison of anthropometric indices in the study participants before and after intervention

Variables	Ginger group (*n* = 20)	Placebo group (*n* = 21)	Mean difference (CI 95%)	*P-value*
**Weight (kg)**				
Baseline	69.67 ± 10.76	74.55 ± 14.31	-4.88 (-12.58, 2.85)	0.209†
Endpoint	69.79 ± 10.38	74.36 ± 15.21	-0.02 (-0.92, 0.88)	0.965††
Mean difference (CI 95%)	-0.28 (-0.93, 0.36)	-0.12 (-0.77, 0.54)		
P-value*	0.370	0.704		
**BMI (kg/m**^**2**^**)**				
Baseline	26.48 ± 3.71	28.41 ± 4.35	-1.92 (-4.38, 0.54)	0.123†
Endpoint	26.50 ± 3.90	28.40 ± 4.58	-0.14 (-0.36, 0.33)	0.934††
Mean difference (CI 95%)	-0.11 (-0.36, 0.14)	-0.06 (-0.30, 0.18)		
P-value*	0.364	0.614		
**Waist circumference (29)**				
Baseline	99.08 ± 10.14	104.18 ± 10.44	0.53 (-4/71, 3/63)	0.108†
Endpoint	99.05 ± 9.34	104.07 ± 11.3	0.09 (-0.57, 0.75)	0.780††
Mean difference (CI 95%)	-0.34 (-0.80, 0.11)	-0.31 (-0.78, 0.17)		
P-value*	0.133	0.189		
**Hip circumference (29)**				
Baseline	98.88 ± 8.05	101.92 ± 9.48	-3.04 (-8.39, 2.32)	0.258†
Endpoint	98.73 ± 8.01	101.99 ± 9.88	0.06 (-0.28, 0.40)	0.541††
Mean difference (CI 95%)	-0.14 (-0.36, 0.07)	-0.21 (-0.51, 0.09)		
P-value*	0.182	0.167		
**Waist to hip ratio**				
Baseline	1.02 (0.94, 1.05)	1.03 (1.01, 1.05)	-	0.346#
Endpoint	1.02 (0.95, 1.04)	1.02 (1.01, 1.05)	-	0.401#
Mean difference (CI 95%)	-	-		
P-value**	0.477	0.404		
**Physical activity (MET-min/week)**				
Baseline	310.50 (143.75, 594.00)	241 (198.75, 297.00)	-	0.533#
Endpoint	355.50 (161.25, 583.00)	266 (198.50, 323.00)	-	0.308#
Mean difference (CI 95%)	-	-		
P-value**	0.091	0.249		

Values are reported as Mean ± SD or Median (IQR) for quantitative data. *Paired Samples T-Test, †Independent Samples T-Test, #Mann–Whitney U, **Wilcoxon Signed Ranks Test, ††ANCOVA adjusted for baseline values and energy intake

*BMI* Body mass index

### Biochemical parameters

Fasting blood glucose levels in the ginger group were significantly lower at the end of the study as compared to the baseline (*P* = *0.001*), while there were no statistically significant changes in the placebo group (*P* > *0.05*). There was a statistically significant diference between the two study groups in terms of FBG so that a considerable decrease was seen in the ginger group adjusted for baseline values, calorie consumption, insulin intake and weight change, F (1, 35) = 17.954, *p*˂0.001 (Table 5). Similarly, HOMA-IR fell considerably in the ginger group at the end of the trial compared to the commencement of the study (*P* = *0.001*), and between group differences remained significant after adjusting for the baseline values and the other determined confounders F (1, 35) = 7.111, *p* = *0.012*. Of Note, there were no significant diferences in terms of serum levels of insulin within the two study groups (*p* > *0.05*; Table 5).

**Table 5:** Overall metabolic profile of study participants before and after intervention

Variables	Ginger group (*n* = 20)	Placebo group (*n* = 21)	Mean difference (CI 95%)	*P-value*
**FBG (mg/dl) Reference Range (70–115 mg/dl) > 115 mg/dl = Diabetic**				
Baseline	174.59 ± 56.12	150.18 ± 33.98	22.41 (-3.82, 52.64)	0.090†
Endpoint	132.85 ± 33.20	156.71 ± 34.54	-35.71 (-52.82, -18.60)	˂0.001†††
Mean difference (CI 95%)	-34.20 (-52.67, -15.73)	4.95 (-5,57, 15.48)		
P-value*	**0.001**	0.338		
**Insulin (μIU/ml) Reference Range for Diabetics (0.7–25 μIU/ml)**				
Baseline	11.16 ± 1.68	10.53 ± 1.54	0.63 (-0.35, 1.61)	0.199†
Endpoint	10.63 ± 1.47	10.08 ± 1.37	0.26 (-0.55, 1.07)	0.522†††
Mean difference (CI 95%)	-0.45 (-0.96, 0.07)	-0.50 (-1.20, 0.21)		
P-value**	0.084	0.159		
**HOMA-IR > 2.5 = Positive for Insulin Resistance**				
Baseline	4.92 ± 2.17	3.99 ± 1.33	0.93 (-0.17, 2.02)	0.094†
Endpoint	3.53 ± 1.20	3.97 ± 1.25	-0.85 (-1.50, -0.20)	0.012†††
Mean difference (CI 95%)	-1.11 (-1.71, -0.50)	-0.08 (-0.58, 0.43)		
P-value*	**0.001**	0.758		
**Urea (mg/dl) Reference Range Men: 18–55 mg/dl Women: 15–43 mg/dl**				
Baseline	102.86 ± 30.45	97.73 ± 20.75	5.15 (-11.01, 21.31)	0.517†
Endpoint	92.03 ± 26.32	104.10 ± 22.91	-14.73 (-29.05, -0.42)	0.028††
Mean difference (CI 95%)	-14.82 (-26.71, -2.92)	6.38 (-2.32, 15.09)		
P-value*	**0.017**	0.142		
**Cr (mg/dl) Reference Range: (0.6–1.4 mg/dl)**				
Baseline	8.75 ± 2.07	8.06 ± 1.73	0.69 (-0.47, 1.85)	0.237†
Endpoint	8.05 ± 1.95	8.14 ± 1.60	-0.32 (-1.54, 0.91)	0.600††
Mean difference (CI 95%)	-0.94 (-1.80, -0.08)	0.08 (-0.61, 0.77)		
P-value*	**0.034**	0.805		
**PAB (HK)**				
Baseline	48.66 ± 17.61	49.91 ± 20.23	-1.25 (-12.78, 10.29)	0.828†
Endpoint	43.81 ± 16.37	51.42 ± 22.22	-7.58 (-16.82, 1.66)	0.105††
Mean difference (CI 95%)	-4.98 (-8,76, -1.20)	1.52 (-7.06, 10.10)		
P-value*	**0.013**	0.716		

Values are reported as Mean ± SD or Median (IQR) for quantitative data. * Paired Samples T-Test, † Independent Samples T-Test, ** Wilcoxon Signed Ranks Test, ††† ANCOVA adjusted for baseline values, Insulin intake, calorie intake, and weight, †† ANCOVA adjusted for baseline values, calorie intake, and weight

*FBG* Fasting blood glucose, *HOMA-IR* Homeostatic model assessment of insulin resistance, *Cr* Creatinine, *PAB* Prooxidant–antioxidant balance, *HK* Hamidi-Koliakos Arbitrary Unit Based on the Percentage of Hydrogen Peroxide Evaluated in Standard Solution

**Fig. 2:**
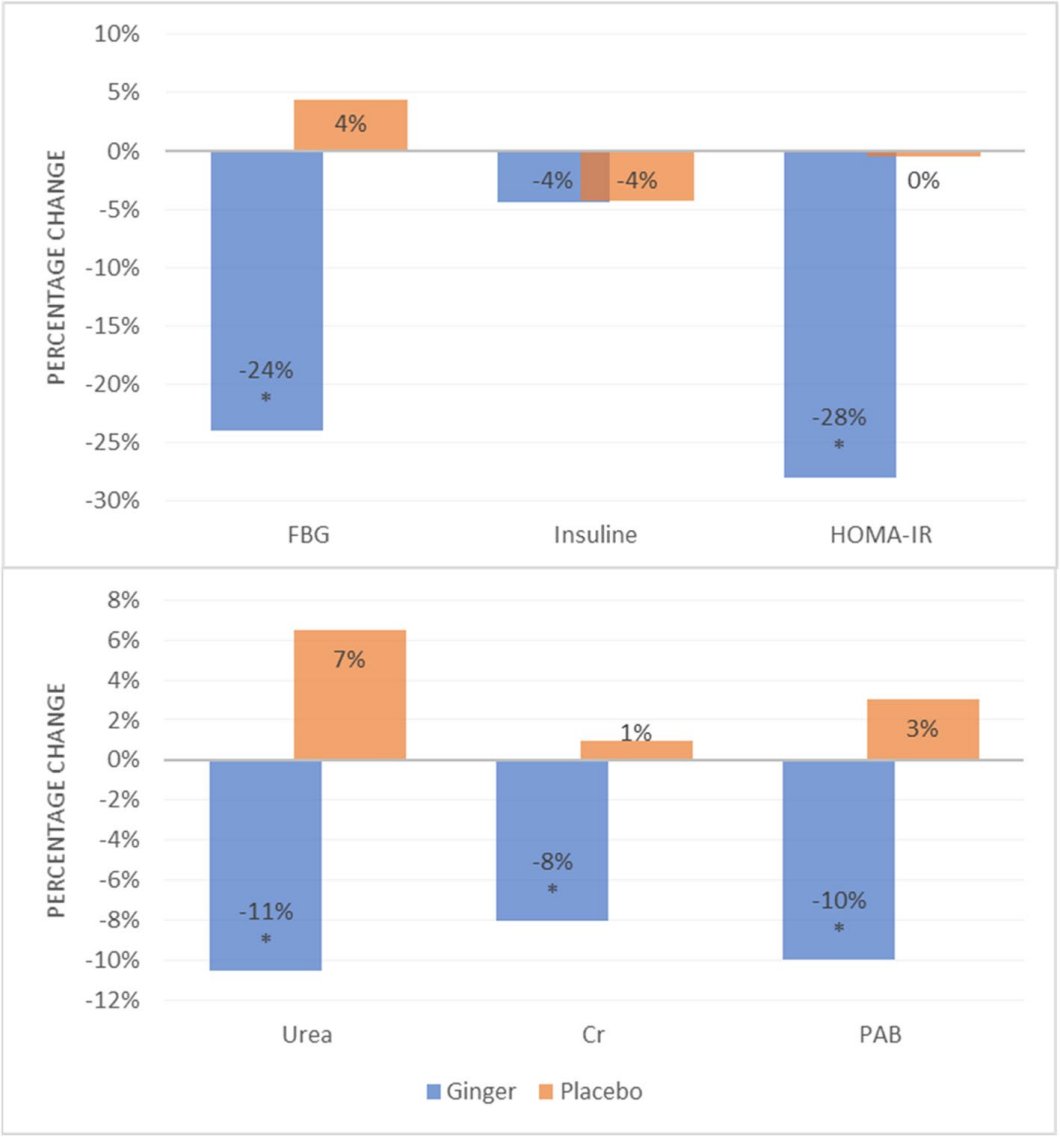
Mean percentage change in the metabolic profile of study participants in the ginger and placebo groups

**Fig. 3:**
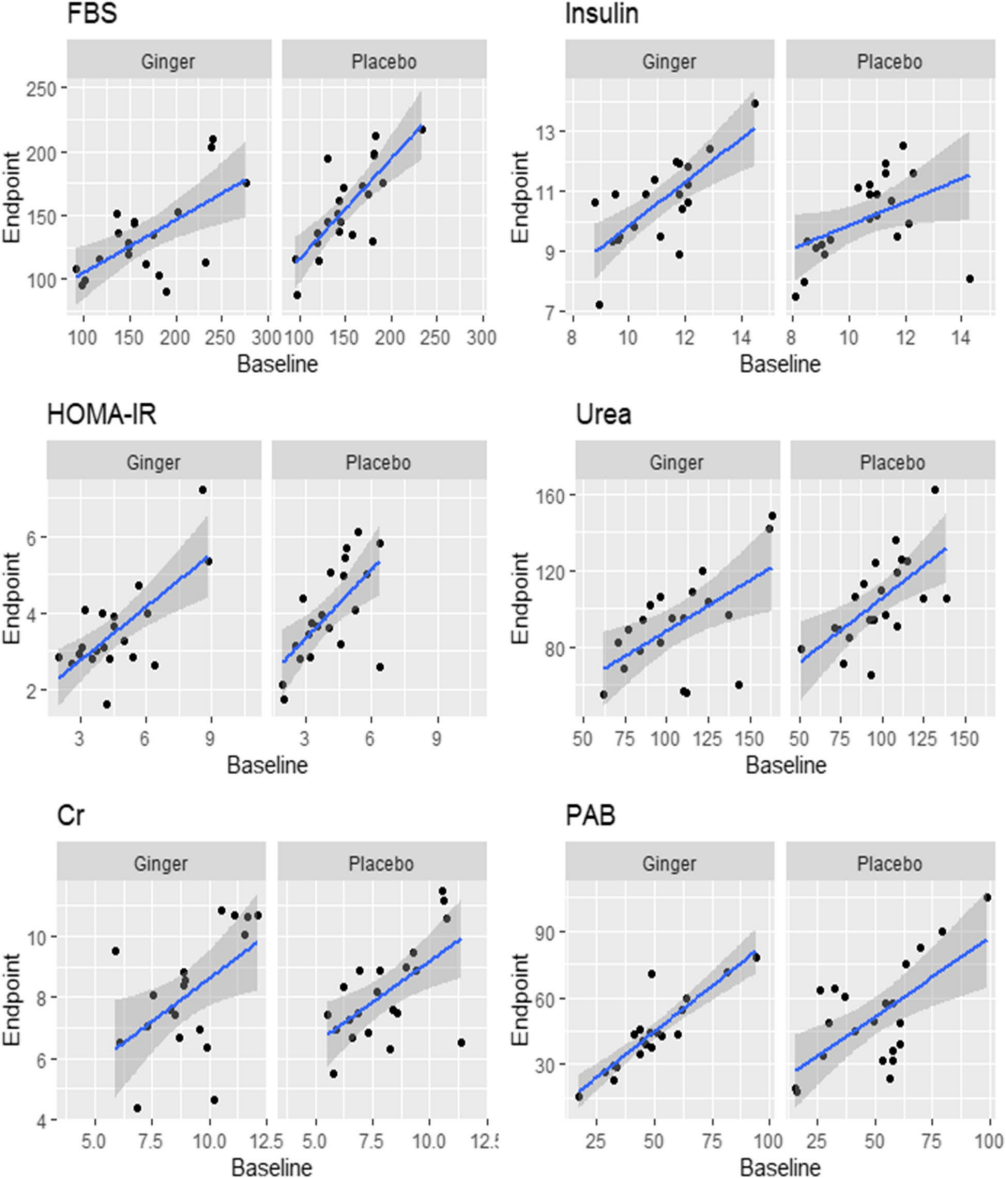
The slopes of the lines of best fit for each group (ginger and placebo) are based on ANCOVA

The ginger group showed remarkable reductions in urea and creatinine concentrations in the blood, however, the differences of serum levels of urea remained statistically significant after adjusting for the aforementioned covariates, F (1, 36) = 5.323, *p* = 0.028 (after adjusting for variables); however, this was not observed in the case of creatinine (*p* > *0.05*; Table 5). Despite the fact that there were no statistically significant diferences in serum PAB between the two groups, It decreased dramatically in the ginger group (*p* = *0.013*; Table 5). The average change percent of the metabolic biomarkers of the participants is shown in Fig. 2. Besides, the slopes of the lines of best fit for each group (ginger and placebo) based on ANCOVA are shown in Fig. 3.

### Harms

Some mild gastrointestinal complications, like heartburn were reported, however, it did not lead to the withdrawal of any participants from the study.

## Discussion

To the best of our knowledge, this is the first clinical trial aimed to investigate the effects of ginger supplementation on diabetic ESRD patients undergoing hemodialysis. According to current findings, short-term ginger supplementation for eight weeks in this group of patientsmay improve glycemic control. Besides, it mar have beneficial effects on some renal function parameters. Furthermore, the results showed no significant changes in energy, macronutrients, and micronutrient intake between the two groups at the end of the study compared to the beginning, indicating the diet was not a notable confounder in this study.

Blood glucose control in diabetic patients, particularly those on hemodialysis, may be difficult under the shadow of of hypoglycemia. Moreover, these patients' treatment choices are limited due to impaired renal function and the buildup of medication metabolites in the body [27]. Current results on the efficacy of ginger in lowering FBG and HOMA-IR (by 24% and 28%) are in line with the previous research [16]. The coefficient of variation (CV%) relative to the mean for FBS is 32% in the ginger group versus 23% in the placebo group. Also, the calculated ranges (i.e., minimum and maximum) in the ginger and placebo groups at the baseline are 185 and 135, and at the endpoint, they are 120 and 129, respectively. In this regard, we used our randomization process to make the group similar by stratifying on FBS categories. Afterward, we adjusted the dissimilarities between groups in the multivariable statistical modeling, ANCOVA. Based on our literature review, only one study was conducted to assess the hypoglycemic effect of ginger in ESRD patients [16], which indicated the positive effects of ginger in reducing FBS; besides, some animal studies demonstrated that ginger decreased blood glucose levels in kidney dysfunction [28–32]. No significant impact on serum insulin levels was observed in the present study that was in accordance with the study done by Mozaffari-Khosravi et al., showed no significant effect of ginger on serum insulin levels in diabeticpatients [33]. Mahluji et al., on the other hand, discovered that eating two grams of ginger per day for two months in patients with type 2 diabetes had no impact on FBG but may lower blood insulin and insulin resistance [34].

The phenols, polyphenols, and flavonoids in ginger are thought to have hypoglycemic effects [35]. Ginger seems to help with insulin resistance by increasing GLUT4 translocation from the cytosol to the cell membrane [36]. Besides, ginger's inhibition of the hepatic glucose 6-phosphatase enzyme activity may lower blood glucose levels [37]. While studies on the effect of ginger supplementation on insulin levels in the blood have produced mixed results [38], some active constituents of ginger, such as 6-gingerol and 6-shogaol, may affect insulin resistance by upregulating adiponectin and peroxisome proliferator-activated receptor, which improves insulin sensitivity and glycemic control [39]. It has been established that the PPAR-γ agonists increase plasma levels of adiponectin in diabetes [40], and adiponectin enhances insulin sensitivity by reducing inflammation and oxidative stress [25, 41]. Hence, ginger may have beneficial effects on maintaining the homeostasis of glucose.

In this study, ginger reduced urea levels consistent with prior animal studies [42–44]. This suggests that ginger may have some mild renoprotectve effects in diabetic patients with ESRD; however, there are very few human studies to support this claim. In this study, serum creatinine levels decreased by 8% in the ginger group, but the differences did not reach a significant level.These findings were consistent with some animal studies [31, 45, 46]. in which, the administration of ginger caused to lower serum creatinine levels [43, 47, 48]. This disagreement may be due to the differences in the amount and form of ginger administered in animal studies.

The renoprotective properties in lowering the serum levels of urea may be attributed to polyphenols and flavonoids in ginger [49]. In earlier studies, free radicals have been linked to renal failure in various ways [18, 50]. Ginger improves kidney function by scavenging free radicals [51]. According to Uz et al., ginger administration raised the levels of several antioxidant enzymes (serum superoxide dismutase (SOD) and glutathione peroxidase (GPx)) that defend against oxygen free radicals in rats suffering from Renal Ischemia/Reperfusion damage [18]. Urea can lead to functional changes in the kidney by increasing the production of free radicals and apoptosis [52]. While creatinine is filtered at the glomerulus and eliminated from the plasma by the kidneys, urea reabsorption is hypothesized to occur as a consequence of water reabsorption [53]. Therefore, ginger might have influenced creatinine excretion and urea reabsorption in the nephrons.

In a live organism, there are several antioxidant and oxidant factors, and a disturbance of the pro-oxidant/antioxidant equilibrium may result in tissue harm. The pro-oxidant antioxidant balance showed substantial promise for mortality prediction in individuals with chronic renal disease, and oxidative stress in diabetic patients were much higher than in healthy individuals [54–56]. According to the current study, ginger caused lowering serum levels of PAB by 10%, although the differences between the two study groups were not statistically significant. The presence of polyphenols and flavonoids in ginger is thought to be responsible for its antioxidant properties [57]. No study was found about the effects of ginger on serum levels of PAB in patients undergoing hemodialysis. However, two human studies have been conducted on the effect of ginger on malondialdehyde (MDA) levels in patients with ESRD, and their results were contradictory. Although Imani et al. observed no effect of ginger on serum levels of MDA in peritoneal dialysis patients, ginger caused lower MDA levels in ESRD patients in the Seddik et al. study [16, 58].

Hyperglycemia increases the production of Reactive oxygen species (ROS), and ginger can reduce ROS levels by lowering serum blood glucose concentrations [42]. In addition, the antioxidant activity of ginger could be explained through the following possible mechanisms: 1) Increasing nuclear factor erythroid-related factor 2 (Nrf2) signaling by ginger's bioactive compounds (such as 6-shogaol) [59]. 2) Inhibition of protein kinase C [60]. 3) Inhibition of the polyol pathway [61]. 4) Reducing the production of advanced glycation products (AGEs) can reduce these compounds' destructive effects in increasing ROS production [62].

The results of the present study indicated that diabetic ESRD patients undergoing hemodialysis who were 18 years of age or older and with no defined history of acute gastrointestinal diseases, thyroid abnormalities, gallstones, or ginger products sensitivity may benefit from ginger supplementation. This study was the first one that evaluated the PAB in kidney disease patients. Moreover, a high percentage of patients' adherence to the study protocol can be considered as another study's strength, indicating negligible side effects of ginger in this group of patients. We did not assess glycosylated hemoglobin, components of the body's antioxidant system such as antioxidant enzymes of SOD, GPx, catalase, serum levels of MDA, and some indicators of the body's oxidant status in urine due to financial constraints. Moreover, it seems that the study's short supplementation duration of eight weeks and the small number of patients included in the study were another drawbacks and the causes of certain non-statistically significant alterations at the conclusion.

## Conclusion

In this study, ginger could result in lower blood glucose levels, enhanced insulin sensitivity, and lower serum urea levels with no effect on prooxidant-antioxidant balance (PAB) in patients with diabetes and ESRD who were receiving hemodialysis. However, further studies with a more extended intervention period and various doses and formes of ginger are needed.

## Data Availability

The data that support the findings of this study are available from [Zohreh Ghoreishi] but restrictions apply to the availability of these data, which were used under license for the current study, and so are not publicly available. Data are however available from the authors upon reasonable request and with permission of [Zohreh Ghoreishi].
